# Transactions of the Chicago Society of Physicians and Surgeons, March 23d, 1874

**Published:** 1874-04-01

**Authors:** Plym. S. Hayes


### Society Reports.

### TRANSACTIONS OF THE CHICAGO SOCIETY OF PHYSICIANS AND SURGEONS.

### MEETING OF MARCH 23D, 1874.

*Reported by Plym. S. Hayes, M.D.*

THE Society met as usual, in the parlor of the Grand Pacific Hotel, the President in the chair.

The minutes of the preceding meeting were read and approved.

Dr. C. T. Parkes was unanimously elected to membership; and the names of Drs. Ralph E. Starkweather, W. H. Warn, and E. P. B. Wilder, were presented as candidates.

Dr. F. H. Davis then read a clinical report of operations on the mouth, which described Dr. I. Manly's method of rendering the hæmorrhage less profuse, and at the same time continuing the anæsthesia.

Dr. Henrotin then exhibited an enlarged heart, and related the following case: The patient, a laborer, of foreign birth, and in previous good health, with the exception of an attack of pneumonia a year prior to his death; temperate; weighing about one hundred and seventy pounds — fell down when at work, and died in three minutes. He had been able to sleep in any position; had not had dyspnœa; and there was no dropsical effusion, except in the pericardium. He had not been at work since last fall, but had been engaged in labor—the first time for months—only about two hours previous to his death. Upon opening the chest, the pericardium was found enormously distended, and pushed forward into the opening. This was punctured, and about fifteen ounces of clear serum evacuated. The pericardium showed no signs of inflammation. The heart, enormously enlarged, was then uncovered. Its right side was distended with dark fluid blood, and the right auricle was so large that it resembled a tumor. When opened, and measured transversely, the auricular walls were found to be ten inches in extent. The aortic semilunar valves were rigid and fixed, from a cretaceous deposit. There were no heart-clots; nor were there any clots found in the large arteries. The lungs were congested. The heart weighed thirty-two ounces.

A discussion then followed as to the direct cause of death, in which Drs. Henrotin, Hyde, Lyman, and others, participated.

Dr. Bartlett then read the Annual Report of the Section on Pathology; the sub-sections on the pathology of the nervous system, syphilis, and cutaneous affections, making separate reports. All that was new or interesting in this branch of science, published in the medical periodicals of the past year, was collected and presented.

The Secretary read a communication from Dr. Quine, on the part of the Committee of Arrangements, in regard to entertaining the State Medical Society, which convenes in this city in May next. It was resolved, on motion of Dr. Hyde, that "this Society desires to co-operate with, and extend all requisite aid to, the Committee of Arrangements in providing an entertainment for the State Medical Society."

In furtherance of Dr. Owen's motion, that every member of the Society connected with the Staff of a hospital be added to the Committee on Clinical Reports, which was adopted at the meeting of March 9th, a list of names of those added to the Committee was read.

The Society then adjourned.

